# Unhealthy Dieting During the COVID-19 Pandemic: An Opinion Regarding the Harmful Effects on Brain Health

**DOI:** 10.3389/fnut.2022.876112

**Published:** 2022-04-28

**Authors:** Iván Rentería, Patricia Concepción García-Suárez, José Moncada-Jiménez, Juan Pablo Machado-Parra, Barbara Moura Antunes, Fabio Santos Lira, Alberto Jiménez-Maldonado

**Affiliations:** ^1^Facultad de Deportes, Universidad Autónoma de Baja California, Ensenada, Mexico; ^2^Department of Health, Sports and Exercise Sciences, University of Kansas, Lawrence, KS, United States; ^3^Human Movement Sciences Research Center (CIMOHU), University of Costa Rica, San José, Costa Rica; ^4^Exercise and Immunometabolism Research Group, Department of Physical Education, Paulista State University, UNESP, Presidente Prudente, São Paulo, Brazil

**Keywords:** COVID-19 pandemic, mental health, brain function, nutrition, brain

## Abstract

Since 2020, the world has been suffering from a pandemic that has affected thousands of people regardless of socio-economic conditions, forcing the population to adopt different strategies to prevent and control the advance of the disease, one of which is social distancing. Even though social distancing is a safe strategy to reduce the spread of COVID-19, it is also the cause of a rising sedentary behavior. This behavior develops an excess of fat tissue that leads to metabolic and inflammatory disruption related to chronic diseases and mental health disorders, such as anxiety, depression, and sleep issues. Furthermore, the adoption of dietary patterns involving the consumption of ultra-processed foods, higher in fats and sugars, and the reduction of fresh and healthy foods may play a role in the progress of the disease. In this perspective, we will discuss how an unhealthy diet can affect brain function and, consequently, be a risk factor for mental health diseases.

## Introduction

The COVID-19 outbreak started in March of 2020; this human threat substantially modified the lifestyle of people around the world. Quarantine and social distancing were the two well-known initial preventive care measures imposed by governments worldwide to minimize the spread of infection of COVID-19. Because of the fast virus spread, schools were closed, national and international travel was restricted or forbidden, and other social activities, such as amateur and professional sports tournaments and musicals, were canceled. In addition, hundreds of countries kept their population in lockdown at home in isolation indefinitely to reduce the risk of transmission of the COVID-19. Although social distancing is a safe strategy to reduce the spread of the COVID-19, the lockdown increased sedentary behavior [might be defined as an energy expenditure of ≤1.5 metabolic equivalents of task (METs)] (1), mental and physical health problems (anxiety, depression, and others) (2, 3), and sleep and circadian rhythm disruption in the population (4, 5). The latter impacts body composition by promoting the greater intake of high energy-dense food types (5, 6). On the other hand, circadian misalignment can be achieved by alteration of the sleep and feed patterns (specially the increase in high-fat food intake), and potentially leading to cardiovascular disease (7). Furthermore, circadian clock genes trigger the onset of metabolic disorders, including metabolic syndrome (MetS) (7, 8).

The gain of excessive adipose tissue leads to local and systemic pro-inflammatory conditions, impairing glucose metabolism, and the onset of metabolic disorders (e.g., type-2 diabetes -T2D-), altering the functionality of organs and systems evenly. Moreover, the pro-inflammatory state *per se* harms the structure of brain topological integration and function. Similarly, poor diet quality, defined as the diet with a reduced variety and nutritional deficiency, does not align with international guidelines (9), represents another factor that generates dysfunctional brain activity (10). Unfortunately, during the COVID-19 lockdown, the population adopted unhealthy diet patterns from previous bad habits or eating behaviors or by emerging social conditions (e.g., reduction of income as a direct consequence of a sharp raising in the unemployment rate) (11, 12). Thereby, scientists and international organizations recommended maintaining a healthy diet focusing on strengthening the immune system and coping with the COVID-19 infection (13–15). However, as mentioned above, an unhealthy diet is a factor that negatively affects brain function. Therefore, the present perspective article briefly discusses how a current poor diet in the population during the COVID-19 lockdown might affect brain health.

## Ultra-Processed Food-Based Diet: A Risk Factor for Brain Dysfunction During COVID-19

According to information provided by international organizations and scientists, ultra-processed foods (UPFs) have undergone excessive industrial manufacture. As a result, UPFs are deficient in dietary fiber, protein, and micronutrients, these products contain little to no whole foods, (16–18). Furthermore, UPFs are energy-dense products that contain artificial components that modify textures, flavors, and colors, producing palatable and more attractive foods (17). The UPFs are typically ready for consumption like soft drinks, sugar drinks, fatty or salty snack products, ice cream, French fries, burgers, desserts, and more products offered as a whole variety of fast foods (16, 17, 19).

The excessive consumption of UPFs is considered the primary source of non-communicable diseases (i.e., obesity, MetS, T2D, etc.) (19). In addition, during the COVID-19 lockdown, individuals have reported higher UPFs consumption in contrast to pre-pandemic times (20–24).

Currently, some studies pointed out the excess of dietary fats can promote changes in gut-microbiota and favor augmented lipopolysaccharides (LPS) extravasation to blood (25). Augmented LPS in blood lead to Toll-Like Receptor 4 (TLR-4) activation via binding the cellular membrane, stimulating pro-inflammatory signaling cascades, increasing cytokine synthesis (TNFα, interleukin -IL- 1B, IL-6, and interferon γ –IFNγ-). This constant cycle (higher dietary fat intake and blood LPS) favors the development of chronic metabolic disruptions, like insulin resistance (26). Recently, Teixeira et al. (27) demonstrated an increased microbial translocation and hyper inflammation in patients with severe COVID-19, provoking higher monocyte activation, which may be associated with worsening outcomes, including death.

Linked with the preponderance to UPFs ingestion, the SARS-Cov2 virus directly and indirectly affects at-risk populations (e.g., hypertensive patients, aged people). Social distancing has also caused the world population's physical activity reduction (2) both lifestyle habits induce body weight gain. In agreement, recent work reported that obesity prevalence has raised during the ongoing social distancing (28, 29). In obesity, immune, adipose tissue, skeletal muscle, and liver engage in a particular crosstalk leading to IR (30–38).

There is evidence that IR leads to hyperglycemia and a parallel increase in pancreatic β-cell insulin secretion (i.e., hyperinsulinemia) (39, 40). These conditions often lead to a cascade of metabolic risk factors collectively referred to as MetS, characterized by central obesity, IR, dyslipidemia, and hypertension (41), and it is known to increase T2D risk by over 2-fold (42–44). Contrary to MetS, T2D is mainly impaired insulin secretion resulting from IR (42).

Besides the pathological effects of MetS and T2D on peripheral organs, recent evidence also suggests a negative impact on brain function and surrounding areas (45–48), such as the blood-brain barrier (BBB) (49–52). The BBB regulates the molecular exchange between the peripheral blood and the brain (53, 54). Conceptual models suggest that chronic peripheral inflammation due to T2D and MetS increases the BBB permeability to leucocytes and external molecules into the brain (41, 42). Thus, the cerebral response begins with an inflammatory response (43, 44), followed by a pro-inflammatory response that alters endothelial cells (ECs), increasing the BBB permeability (55, 56). Other studies show that T2D increases the inflammatory profile of ECs and BBB permeability, a response closely associated with cognitive impairment (57, 58).

In addition, MetS depicts elevated serum triacylglycerol (TGs) and low high-density lipoprotein (HDL-c) concentrations (33). Nevertheless, cross-sectional studies have reported equivocal findings regarding the association between high serum TGs and cognitive function in humans (59, 60). Some authors report an adverse effect of TGs on cognitive function (46, 60), while others suggest a positive outcome on brain function (45). In this regard, it is worth indicating that the former study was in a Chinese sample, whereas the other studies were in the western populations. This evidence suggests racial/ethnic disparities in the effects of TGs on cognitive function. Finally, IR in the brain induced by TGs was also demonstrated (47).

**Figure 1:**
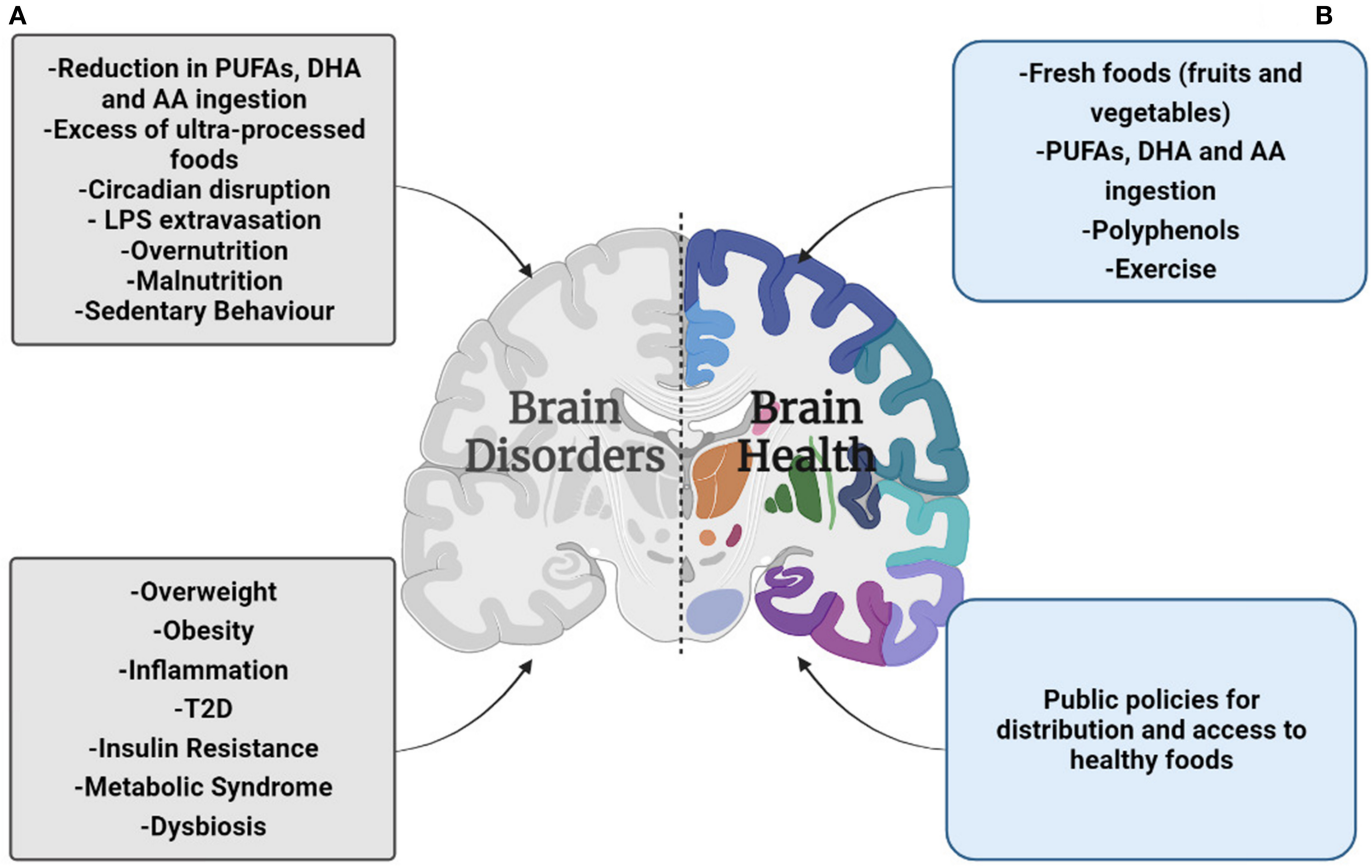
Summary overview of the malnutrition effects on brain health during the COVID-19 pandemic. **(A)** The preponderance by the UPF's ingestion, circadian disruption, and a sedentary lifestyle will facility the gain of body weight, leading to suffering overweight/obesity, and in a worst-case scenario suffer metabolic syndrome and T2D. Moreover, overweight/obesity increases the risk of low-grade chronic inflammation; the pro-inflammatory cytokines hinder the blood-brain barrier function deteriorating brain health. Additionally, the higher rate of unemployment and the rising price of food have reduced the affordability of fresh products, and protein based-products, resulting in a poor quality diet. This condition is a factor that reduces brain integrity that leads to suffering brain disorders. **(B)** The public services focused to facilitate the practice of physical exercise during the lockdown, and better distribution and access to healthy food will strengthen the brain health and reduce the risk to suffer brain disorders.

Another concern in MetS is the continuous hyperglycemic state that facilitates the non-enzymatic interaction between glucose and proteins (48, 49). Glycated protein generates advanced glycation end-products (AGEs). These molecules have been associated with reduction in BBB integrity (39, 49). Moreover, AGEs activate the synthesis of pro-inflammatory cytokines in the BBB's ECs, causing a pro-inflammatory feedback loop (42). Chronic hyperglycemia triggers several metabolic signaling mechanisms that induce inflammation, apoptosis, and the synthesis of reactive oxygen species (ROS) (50). Additionally, studies performed in obese rodents show that ROS increases BBB permeability, reducing the expression of proteins associated with tight junctions (37, 51). On the other hand, hyperglycemia *per se* harms the brain in patients with T2D (31, 52, 53). At least two mechanisms negatively contribute to this: 1) hyperglycemia is associated with brain atrophy (53), 2), hyperglycemia increases the risk for stroke, leading to brain acidosis (52, 54). Furthermore, hyperglycemia increases the activity of excitatory neurotransmitters (e.g., glutamate), resulting in a higher calcium concentration in neural cytosol to induce cell death (54). Finally, hyperglycemia is a factor that reduces the topological integration in brain (45), which possibly contributes to cognitive impairment in T2D (45) (Figure 1).

## Poor Diet Quality: A Risk Factor for Brain Integrity During COVID-19

The human body is a structure that requires energy for anabolic and catabolic processes. In this sense, ingesting food was initially considered a survival practice. However, anthropologists proved hypotheses concerning the diet role in Hominids evolution (61–65). For instance, cooking foods improved digestion capacity (62, 66). Additionally, cuisine foods increased the availability of the nutrients present in plants and meat (66). The previous conditions and others facilitated the brain evolution (i.e., encephalization) (62, 67). Therefore, the diet components have played a relevant role throughout the Homo evolution in conjunction with food processing. In this sense, fatty acids, mainly the long-chain polyunsaturated fatty acids (PUFAs), docosahexaenoic acid (DHA), and arachidonic acid (AA), have provided essential effects on brain evolution (63, 68). Moreover, AA is a lipid that strengthens synaptic transmission (69, 70). However, the AA is also a precursor of molecules linked with inflammatory responses such as prostaglandins and pro-inflammatory cytokines, such as TNFα and IL-1B (71, 72). Another example of dietary adaptations is the lactose tolerance of some populations. The latter is due to milk consumption after the weaning period, keeping the lactase enzyme active (73). As can be noted, the diet helps humans deal with the context of living, allowing us to say that we are what we eat. Therefore, the population who show a poor-quality diet will have few tools to cover all the surrounding challenges.

The COVID-19 lockdown disrupted the dietary patterns in the world population (74, 75), affecting low-and-middle-income countries (11, 12, 76, 77). Therefore, besides the enhancing effect on the UPFs consumption, the COVID-19 outbreak reduced food security, and consequently, the dietary quality (11, 12, 75, 78, 79). Food security is a complex phenomenon that implicates time, physical and economic access to sufficient healthy food to satisfy the nutritional needs and food preferences for a healthy lifestyle (80). The opposite condition is known as food insecurity (FI) (80, 81), which is related to malnutrition (i.e., undernutrition and micronutrient deficiency) (81). During the COVID-19 lockdown, unemployment growth and increased food prices were the main factors reducing food affordability (11). In addition, the lockdown restrictions reduced the food supply chain (78), which in turn reduced the ingestion of fresh products such as fruit and vegetables (75). Together with the prior information, other authors have reported that the population with high FI scores showed higher anxiety levels (78), independent of the socio-economic factors (82). Besides the FI, an inverse relationship is reported between quality diet and anxiety levels in individuals undergoing lockdown (29).

The nutrient deficiency intake might impact brain function. For instance, the PUFAs role on neural membrane integrity, gray matter, and hippocampal volume (83), makes them an important nutrient whose low ingestion contributes to a reduced brain plasticity (83–85). Together with lipid actions, other molecules are also essential to strengthen brain function. Concretely, polyphenols found in fruits and vegetables also have positive effects (86). Resveratrol for example, a phytoalexin present in grapes, berries, tomatoes, nuts, and cocoa (87), demonstrated positive effects on brain function and structure (88, 89). Chronic consumption of resveratrol led to a better cognitive performance (i.e., improving memory) and mood in postmenopausal women (89), the hypothetical mechanism explaining these responses was a better cerebral perfusion modulation in the participants (89). A similar effect was reported in healthy men (90); moreover, this polyphenol enhances the functional connectivity from the hippocampus to frontal, parietal, and occipital areas, improvement in the memory retention correlated with a topological shift in brain, and glucose metabolism in healthy older adults (91).

Conversely, protein malnutrition (PMN) is a risk factor for neuroinflammation and oxidative stress (10, 92, 93). Moreover, the PMN in pregnant women affects brain development and cognition considerably in the offspring (10). The previous findings emphasize the impact of the diet on brain integrity during the COVID-19 outbreak (Figure 1).

Poor diet, nutrient availability, and quality will also impact the gut microbiome and, eventually, brain health. Intestinal content and the brain represent a dynamic bidirectional communication described as the “gut (microbiota)-brain axis” (94). The human gut microbiome includes different types of bacteria responsible for several functions such as energy metabolism, immunity, vitamin synthesis, hormone, and neurotransmitter production, and it also influences human behavior (94–96). Environmental factors (e.g., diet changes caused by the COVID-19 pandemic, medication, exercise) can potentially change the gut microbiome rapidly. In addition, special conditions might lead to a microbial imbalance (i.e., dysbiosis), a factor contributing or associated to the development of some diseases like inflammatory bowel disease (97), atopic diseases (e.g., eczema, asthma, food allergies) (98), type-1 diabetes (99), schizophrenia, and other cognitive disorders (94, 100, 101). Changes in the gut microbiome have shown concomitant changes in brain structure, function, and behaviors (e.g., stress, anxiety, depression) (94, 101). Indeed, the effect of gut microbiome diversity on brain function is partially accounted for by vitamin-mediated neuronal function, neurotransmitter composition, and short-chain fatty acid (sCFas) metabolites (94, 102).

Previous evidence suggests that vagal afferent sensory neurons are microbiota-mediated, regulating information transmission through the kynurenine pathway (103). Probiotic supplementation impacts the central nervous system, and research has shown its effects on anxiety disorders (i.e., anxiolytic effect) (102, 104). Although specific probiotic species affecting brain health are currently under study, recent evidence suggests that anxiety and depressive disorders correlate to higher pro-inflammatory species and lower abundance of sCFas-producing species (105).

## Conclusions and Final Remarks

Social distancing was a strategy implemented worldwide by several governments to reduce the risk of COVID-19 infection. However, this outbreak has impacted the household economy considerably, reducing food affordability and, consequently, the food quality. Although different documents highlight the diet's relevance to strengthening the immune system, there is a lack of emphasis on the diet's role in maintaining brain integrity and functionality during the COVID-19 outbreak. In the current work, we discussed how overweight and obesity impact brain function. Even though this effect is widely reported, the social distancing during COVID-19 increased the risk of suffering obesity. We also discussed how undernutrition is a condition with deleterious effects on brain integrity. Although the mobility restriction is less severe today, the economic impact of the COVID-19 pandemic is still present in society; furthermore, it is projected to reach the pre-pandemic levels until 2023 (106). This scenario impairs individuals' availability to secure adequate nutrients and causes changes in the gut microbiome, resulting in vulnerable brain health and increasing the risk of suffering anxiety, cognitive deficiency, mental disorders, and impaired mood. Finally, different authors indicated that the brain is directly and indirectly affected by COVID-19 (107–109). Therefore, we believe that a fragile brain resulting from malnutrition (i.e., over-nutrition and undernutrition) could worsen the consequences after the COVID-19 infection. Consequently, we consider that governments worldwide must develop strategies to improve the diet quality in the population, mainly during the COVID-19 outbreak. If fulfilled, the possibility of increasing brain health in children, adults, and the elderly is nigh.

## Data Availability Statement

The original contributions presented in the study are included in the article/supplementary material, further inquiries can be directed to the corresponding author.

## Author Contributions

IR, PG-S, and JM-P reviewed the literature, wrote the first draft, and finalized the manuscript. JM-J, BA, and FL finalized the manuscript, BA conceived and designed Figure 1. AJ-M conceived the article focus, reviewed the literature, and wrote the first draft. All authors approved the final version of the manuscript.

## Conflict of Interest

The authors declare that the research was conducted in the absence of any commercial or financial relationships that could be construed as a potential conflict of interest.

## Publisher's Note

All claims expressed in this article are solely those of the authors and do not necessarily represent those of their affiliated organizations, or those of the publisher, the editors and the reviewers. Any product that may be evaluated in this article, or claim that may be made by its manufacturer, is not guaranteed or endorsed by the publisher.
